# Louisville Medical Club

**Published:** 1855-10

**Authors:** 


### LOUISVILLE MEDICAL CLUB.

We regret not to have been able to give in our present issue the Proceedings of the Louisville Medical Club for October. Nothing that we publish, we believe, is read with more interest than the proceedings of this Club, and in future we hope to be able to present to our readers a monthly summary of its transactions.

